# EgoNet: identification of human disease ego-network modules

**DOI:** 10.1186/1471-2164-15-314

**Published:** 2014-04-28

**Authors:** Rendong Yang, Yun Bai, Zhaohui Qin, Tianwei Yu

**Affiliations:** 1 Department of Biostatistics and Bioinformatics, Rollins School of Public Health, Emory University, 1518 Clifton Rd, N.E, Atlanta, GA, USA; 2 Current address: Minnesota Supercomputing Institute for Advanced Computational Research (MSI), University of Minnesota, Minneapolis, MN, USA; 3 Department of Pharmaceutical Sciences, School of Pharmacy, Philadelphia College of Osteopathic Medicine, Suwanee, GA, USA

**Keywords:** Gene expression, Network medicine, Machine learning, Cancer biology, Biological networks, Microarray

## Abstract

**Background:**

Mining novel biomarkers from gene expression profiles for accurate disease classification is challenging due to small sample size and high noise in gene expression measurements. Several studies have proposed integrated analyses of microarray data and protein-protein interaction (PPI) networks to find diagnostic subnetwork markers. However, the neighborhood relationship among network member genes has not been fully considered by those methods, leaving many potential gene markers unidentified. The main idea of this study is to take full advantage of the biological observation that genes associated with the same or similar diseases commonly reside in the same neighborhood of molecular networks.

**Results:**

We present EgoNet, a novel method based on egocentric network-analysis techniques, to exhaustively search and prioritize disease subnetworks and gene markers from a large-scale biological network. When applied to a triple-negative breast cancer (TNBC) microarray dataset, the top selected modules contain both known gene markers in TNBC and novel candidates, such as RAD51 and DOK1, which play a central role in their respective ego-networks by connecting many differentially expressed genes.

**Conclusions:**

Our results suggest that EgoNet, which is based on the ego network concept, allows the identification of novel biomarkers and provides a deeper understanding of their roles in complex diseases.

## Background

Complex human diseases, *e.g.* cancer, diabetes, or autism, are caused by dysregulations of biological networks. Genetic analysis approaches focused on individual genetic determinants are unlikely to characterize the network architecture of complex diseases comprehensively. Creating effective therapies for these diseases requires a thorough understanding of how cells integrate enormous amounts of genomic, proteomic, and environmental information to produce specific cellular functions, and furthermore, how such functions are perturbed in the disease state. Transcriptomics, metabolomics, proteomics and other -omics technologies have the potential to provide insights into complex disease pathogenesis and heterogeneity, especially if they are applied within a network biology framework. “Network medicine” is the rapidly developing field which applies systems biology and network science methods to human disease [1-3].

In the past decade, extensive work has been done to identify differentially expressed genes across different phenotypes, which can be used as diagnostic markers for classifying different disease states or predicting clinical outcomes [4-7]. However, gene markers based on expression data alone are still not reliable [8]. To meet this challenge, many have turned to network medicine to gain a comprehensive understanding of the complex disease process. In contrast to studying individual genes in isolation, mapping human disease-associated genes to interactome data has greatly empowered our understanding of human disease mechanisms [9]. Network-based approaches have multiple potential biological and clinical applications, including a better understanding of the effects of interconnection of disease genes and disease pathways, which, in turn, may offer better targets for drug development. These advances may also lead to more reliable biomarkers to monitor the functional integrity of networks that are perturbed by diseases.

To date, many computational methods have been developed to integrate gene expression profiles with protein-protein interaction maps or pathway databases, with the goal of identifying significant subnetwork markers for predicting biological or clinical outcomes [10-18]. More recently, different machine learning and data mining strategies for feature selection have been applied to identifying a subset of genes that can maximize the prediction performance [19]. Dutkowski *et al.*[20] proposed Network-Guided Forests (NGF) which integrates the key ideas of Random Forests (RF) into the selection of disease modules. However, it involves a random search over subnetworks, leading to possibly different results from different runs with no guarantee of the optimality of the final result. Zhu *et al.*[21] applied network-based Support Vector Machine (SVM) for classification of microarray samples but the method only worked for small subnetworks. More importantly, the above methods are largely heuristic, and the definition of output subnetworks is ambiguous without a formal topological feature. Hence, selected network modules tend to include only significant genes based on their expression profiles, but exclude the non-differentially expressed genes despite the fact that they are functionally linked to many differentially expressed disease genes.

In this study, we developed a novel method called EgoNet to identify significant subnetworks that are functionally associated with diseases, as well as accurately predict clinical outcomes. The type of subnetwork sought by our method is called ego-network, which is well-defined in the study of social networks [22]. In particular, an ego-network is the part of a network that involves a particular node we are focusing on, which we call ego. In addition to the ego, the network consists of a neighborhood including all nodes to which the ego is connected to at a certain path length. The one-step neighborhood contains the nodes the ego is directly connected to (referred to as the ego’s alters), and the links between the ego’s alters. In studying ego-networks, we are interested in examining how egos make use of or are influenced by their alters in terms of associating with disease outcomes. It has been reported that the ego-network played an important role in the inference of novel disease genes and supported predictions in pathogenesis studies [23].

**Figure 1:**
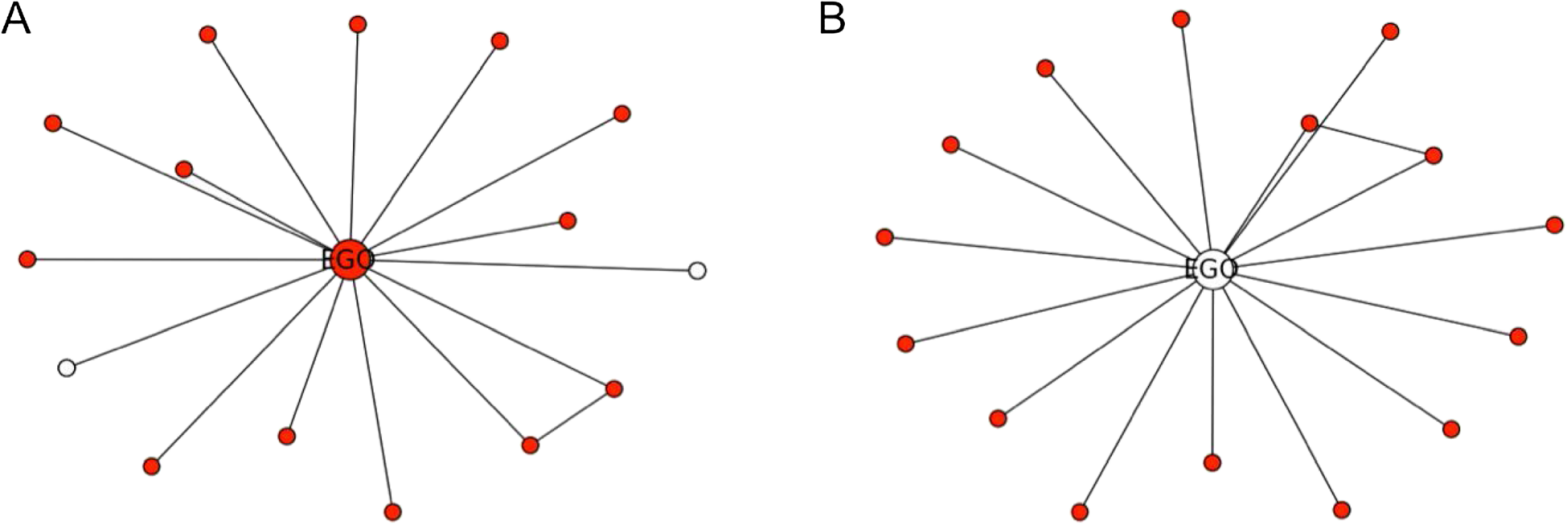
**Two illustrative ego-networks.** Red nodes are putative disease genes, white nodes are hidden disease genes either as alter nodes **(A)** or ego node **(B)**.

The underlying assumption of our model is that if the majority of neighbors of a central disease gene are disease genes, then its other neighbors are likely to be involved in the disease pathway (Figure 1A). Alternatively, if most neighbors of the ego node are associated with a disease, the ego gene itself is considered highly likely to play a role in the disease (Figure 1B). We intend to find the hidden genes that show no significance by themselves but are clustered in a subnetwork module whose genes collectively are highly predictive of the disease status. The ego-network model has been used for network module over-representation analysis in ConsensusPathDB [24]. In this study, we use machine-learning techniques to assess the association between an ego-network with the clinical outcome. This approach allows compensatory effects between the genes in an ego-network, as well as nonlinear relations between the genes and the clinical outcome.

We evaluated the performance of EgoNet in human protein-protein interaction network and a triple negative breast cancer (TNBC) microarray data set. The method not only successfully identified known breast cancer susceptibility genes TP53, BRCA1, BRCA2 from significant ego-networks, but also detected several novel targets, like ABL1 and RAD51 as predictive factors for TNBC patients. We expect that EgoNet can be widely used to infer novel biomarkers for phenotypic outcome prediction of many human diseases.

## Results and discussion

### Overview of EgoNet algorithm

**Figure 2:**
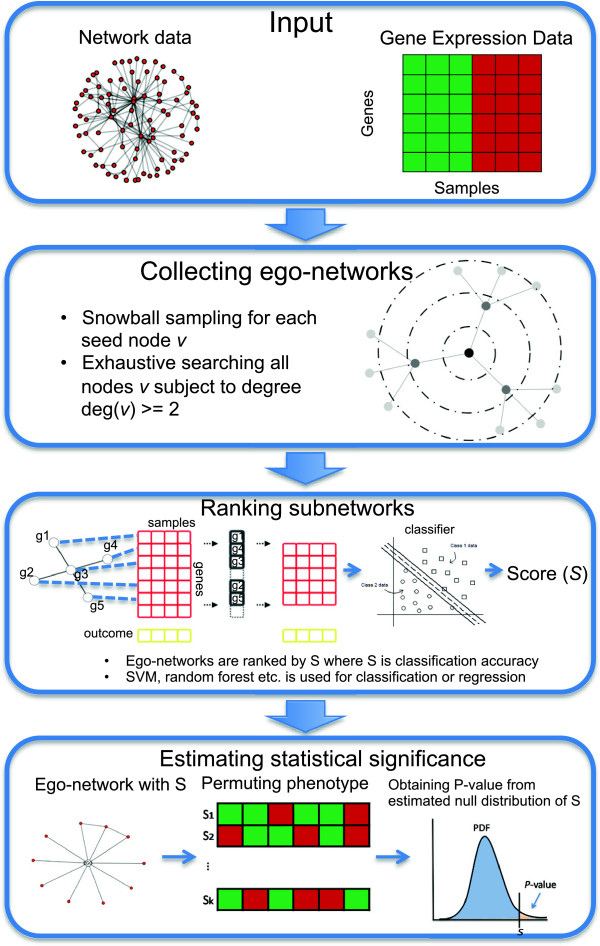
Workflow of the EgoNet algorithm.

The goal of EgoNet algorithm is to identify significant ego-networks from gene expression and large-scale biological network data. As outlined in Figure 2, the algorithm takes the network and gene expression data as input. The input biological network can be a gene regulatory network, a signaling pathway network, or a protein-protein interaction network. The gene expression data needs to be associated with a certain biological or clinical outcome, which can be a categorical, continuous, or survival outcome.

EgoNet iteratively scans through all genes with two or more neighbors in the network. With each initial gene (the ego node), it first finds the score of the level-one ego-network based on how well the genes as a collection predicts the clinical outcome. Then it spreads outward from the ego node progressively to involve more genes in the predictive model. The spreading stops when the prediction accuracy drops (Figure 2; Methods). The above process of growing ego-network is also known as snowball sampling [25]. After obtaining the score of an ego-network, the significance is evaluated by permutation test.

### Simulation studies

To evaluate the capability of an ego network to predict the clinical outcome, a machine-learning method needs to be chosen. In this study, we selected three widely used methods: support vector machines (SVM) [26], K-nearest neighbors (KNN) [27] and random forests (RF) [28], and compared their performance for subnetwork identification through a simulation study.

In each simulation, a scale-free network was generated, and one subnetwork was selected as the ground truth. The subnetwork was linked to the outcome variable through linear or nonlinear relationship. We applied the EgoNet algorithm in conjunction with the three classifiers for subnetwork selection, and inspected if the top identified ego-netowork (s) recovered the true subnetwork. In general, SVM performed the best (Table 1). In both linear and non-linear settings, if we only selected the top ego-netowork in every simulation, SVM successfully recovered the true subnetwork more than 50% of the time. When we increased the number of identified ego-networks to top 5, SVM was able to recover the true subnetwork over 80% of the time. Thus we chose SVM for the subsequent data analysis.

**Table 1:** Percentage of top identified ego-networks successfully matching true subnetworks in simulations using different classification algorithms^*^

	**Top 1**		**Top 5**	
	**Linear (%)**	**Nonlinear (%)**	**Linear (%)**	**Nonlinear (%)**
SVM:	**68**	**53**	89	**83**
RF:	50	42	83	69
KNN:	62	46	**91**	70

^*^Bold numbers denote the best performing method in each simulation setting (column).

Next we compared the performance of EgoNet with the method proposed by Chuang et al. [11], which scores subnetworks using the mutual information between aggregated gene Z-scores and class labels. We simulated two scenarios: (1) All genes in an ego-network, including the ego gene, are associated with the clinical outcome; and (2) All genes in an ego-network, except the ego gene, are associated with the clinical outcome. The second scenario was motivated by our consideration that sometimes a gene functionally related to a disease may not be differentially expressed, while it is surrounded by differentially expressed genes in the network (Figure 1B). In each of the scenarios, we further simulated both linear and nonlinear associations between gene expression and clinical outcome.

The methods were compared in two ways. The first is the accuracy in predicting the clinical outcome, and the second is the rate of correctly recovering the true ego network. For prediction accuracy, we employed the area under the ROC curve (AUC) as the metric to evaluate performance. Additional file 1: Figure S1A shows EgoNet outperformed Chuang et al.’s method in terms of classification accuracy, albeit the difference is relatively small. For true ego network recovery, we calculated the rate of the top selected subnetwork capturing the true ego node. We found EgoNet showed substantially higher proportions of recovering the true ego node (Additional file 1: Figure S1B). As expected, the difference was most pronounced in the scenarios where the ego node itself was not directly associated with the clinical outcome.

### Gene modules differentiate breast cancer subtypes

We applied EgoNet to analyze human PPI network with the expression profiles of the two cohorts of breast cancer patients previously reported by Li et al. [29], which compared the gene expression of 24 sporadic triple negative breast cancer (TNBC) samples against 51 primary breast tumor samples representing all subtypes (NCBI GSE18864). TNBC is characterized by the lack of expression of estrogen receptor (ER), progesterone receptor (PgR), and the human epidermal growth factor receptor 2 (ERBB2, or HER2) [30]. It largely overlaps with the basal-like subtype of breast cancer [31].

The PPI network was obtained from HINT database [32], which collected data from several databases and filtered both systematically and manually to remove low-quality/erroneous interactions. The network contained 8292 human proteins and 27493 high-quality binary physical interactions.

We applied our algorithm to this dataset. We allowed only nodes with more than one connection to serve as egos. From every ego node, we progressively grew the ego-networks by levels, and tested the predictive power. For every ego network, the procedure stopped when the predictive power dropped with the growth. Following this procedure, a total of 5375 ego-networks were examined, and the average of nodes in an ego-network is 30. Since ego-networks spread out in levels, which are the maximum network distance from ego to its alters, we found ~76% of the generated ego-networks were level 1 and ~24% of them were level 2 (Additional file 2: Figure S2). Prediction accuracy for phenotypic outcome of those ego-networks varied between 0.63 and 0.95. We identified the top 50 discriminative ego-networks by setting the accuracy cutoff at 0.9. All were significant with p < 0.001 in permutation tests with 1000 permutations.

**Figure 3:**
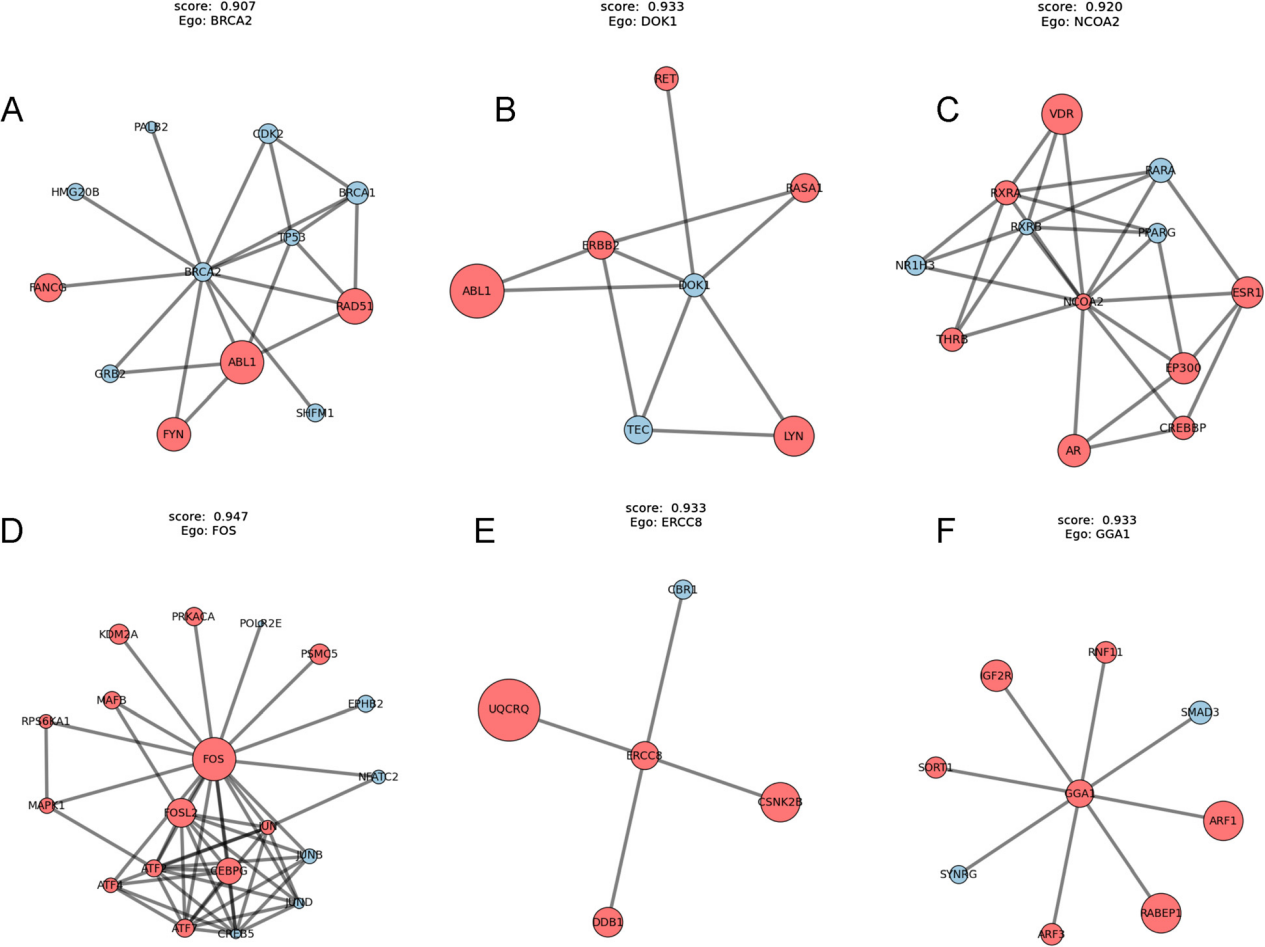
**Identified ego-networks in the TNBC breast cancer dataset.** Module **(A)** contains major breast cancer genes BRCA1, BRCA2 and TP53. Modules **(B)** and **(C)** contain ERBB2 and ESR1 respectively. Examples of other top-scoring modules are shown in **(D-F)**. The area of each node scales with its importance in the classification of the phenotype. Red color indicates differential expression (FDR <0.05 based on a two-tailed t-test with Benjamini & Hochberg FDR adjustment).

BRCA1 and BRCA2 are well-known breast cancer susceptibility genes that belong to tumor suppressor genes [33]. TP53 is a tumor suppressor gene whose mutation is associated with a variety of cancers. Distinct mutation patterns of TP53 was found between the luminal subtypes of breast cancer and TNBC [31]. We explored the three genes in our identified subnetworks. Interestingly, we found they were clustered in one ego-network in which BRCA2 was the ego node (Figure 3A). This observation is consistent with the local property of disease networks – proteins involved in the same disease have an increased tendency to interact with each other [2]. We conducted single-gene level differential expression analysis. At the FDR cutoff of 0.05, none of the three genes showed differential expression between TNBC and non-TNBC breast cancer patients. We further evaluated the importance of each gene on the classification accuracy using a tree-based feature selection algorithm (Method). We found genes with high importance scores were mostly differentially expressed. In the BRCA2 ego-network, breast cancer susceptibility genes ABL1 and RAD51 [34,35] were under such scenario.

The ABL1 proto-oncogene encodes a cytoplasmic and nuclear protein tyrosine kinase that has been implicated in processes of cell differentiation, cell division, and so on [36]. ABL1 is activated into an oncogene and forms a fusion gene with break point cluster (BCR) gene due to missense mutations within the ABL1 kinase domain. The chimeric oncogene BCR-ABL1 has been implicated to play a critical role in the development of chronic myelogenous leukemia [37]. The over-expressed BCR-ABL gene will increase the transmembrane plasma protein expression and constitutively activate the downstream signaling molecules such as Src family kinases [38], including DOK1 and NCOA2, which we discuss below. Thus it is logical to believe that ABL1 is a critical factor in breast cancer development. A detailed examination of the expression level of ABL1 revealed it was substantially over-expressed in TNBC, as compared to other primary breast cancer subtypes (Additional file 3: Figure S3a). Our study suggests ABL1 may be regarded as a predictive factor for differentiating TNBC from other primary breast cancer.

RAD51 encodes the major eukaryotic homologous recombinase [39], which assists in the repair of DNA double strand breaks. The RAD51 protein has been demonstrated to interact with the ssDNA-binding protein BRCA2, a well-known breast cancer susceptibility gene [40]. BRCA2 controls and regulates both the intracellular localization and DNA–binding ability of RAD51 [41,42]. There were some reports suggesting that dysfunctional variants of RAD51 is associated with breast cancer risk. One recent study suggested the association of RAD51 polymorphis with DNA repair in BRCA1 mutation carriers and sporadic breast cancer risk [43]. Smolarz et al. reported that there was a significant positive association between RAD51 polymorphisms and TNBC [44]. In our current study, RAD51 is significantly under-expressed in the TNBC samples (Additional file 3: Figure S3b).

TNBC lacks the expression of three receptors, ER, ERBB2 and PgR [30]. We found two of the corresponding genes from our identified subnetworks, of which ERBB2 was in the DOK1 ego-network (Figure 3B) and ESR1 in the NCOA2 ego-network (Figure 3C). DOK1 is known to be a tumor suppressor gene in epithelial ovarian cancer [45] and lung cancer [46]. It is a substrate of several non-receptor tyrosine kinases [47,48], including breast tumor kinase (BRK) [49]. Since most of DOK1’s alters were differentially expressed, DOK1 may play a role in the molecular pathways of TNBC. DOK1 itself showed a minor under-expression in TNBC (Additional file 3: Figure S3c), though not statistically significant at the FDR level of 0.05. ERBB2 is a member of the DOK1 ego-network. Because the receptor itself is not expressed in TNBC, as expected, the ERBB2 gene was under-expressed in TNBC as compared with other primary breast cancer subtypes (Additional file 3: Figure S3d). ESR1 showed a similar pattern (Additional file 3: Figure S3e).

Our results also suggest NCOA2 could be an important factor in the TNBC gene regulatory pathways. NCOA2, the nuclear receptor coactivator 2, which belongs to the steroid receptor coactivator (SRC) family, has been reported to be broadly involved in many cancers [50]. The SRC family comprises three members, SRC-1 (NCOA1), SRC-2 (NCOA2) and SRC-3 (NCOA3), which are known to be overexpressed in breast cancer and essentially involved in estrogen mediated cancer cell proliferation [51]. Currently, most research on the SRC family has been focused on NCOA1 and NCOA3. Clinical and preclinical studies have demonstrated that overexpressed NCOA1 and NCOA3 are linked to resistance to therapies in breast cancers [52]. For example, overexpression of NCOA3, especially in conjunction with high levels EGF receptor (EGFR) and HER2 (ERBB2), is associated with poor outcome after tamoxifen treatment [53,54]. In ERBB2–overexpressing breast cancer cells, overexpression of NCOA3 also contributes to resistance against the ERBB2 targeting drug transtuzumab [55]. In the current study, NCOA2 is significantly under-expressed in the TNBC samples as compared with other subtypes of primary breast cancer (Additional file 3: Figure S3f). Our results indicate that NCOA2 could be as important as the other two members and play an important role in the TNBC gene regulation.

We shall note that the current study is to compare TNBC with the pool of other subtypes of breast cancer. Thus the resulting sub-networks have more to do with the differences between TNBC and other subtypes, as opposed to directly explaining the clinical characteristics of TNBC itself. Although EgoNet pointed to DOK1 and NCOA2 ego-networks as among the best to separate TNBC from other primary breast cancers, it is still far from establishing a mechanistic explanation. This limitation has to be addressed by future biological studies.

Given an ego-network, a “structural hole” is the absence of an edge among a pair of nodes in the ego network. A well-established proposition in social network analysis is that egos with lots of structural holes are better performers in certain competitive settings [22]. Among our identified ego-networks, we found examples containing few structural holes (Figure 3C-D), and those containing many (Figure 3E-F). The binding mechanism may imply ego genes such as ERCC8 and GGA1 whose ego-networks include many structural holes are key factors to distinguish the TNBC patients.

### Network-based ranking of marker genes

Next, we evaluated the importance of individual genes by considering all the subnetworks together. An important property of disease genes in a molecular network is that the nodes with much higher degrees of linkages, so called hubs, should typically be associated with disease genes [19]. We assume that a putative disease hub is important, and thus should be included in more identified disease subnetworks. For each ego-network, a classification accuracy score is available, and the relative importance values are calculated for genes included in the ego-network. We propose a metric that is the summation of the product of subnetwork score (*S*_*i*_) and node importance (*V*_*ij*_) over all the considered subnetworks, namely

$$M_{j}=\sum_{\left(i=1\right)}^{N}S_{i}V_{\mathrm{ij}},$$

where *i* is the ego-network index, and *V*_*ij*_ is the importance score of the *j*^*th*^ gene in the *i*^*th*^ subnetwork which takes value zero if the gene is not in the subnetwork. Node importance (*V*_*ij*_) is calculated using tree-based feature selection method (Methods).

Table 2 shows the top 20 ranked genes based on their *M* values. We found the list included both differentially expressed (DE) genes and non-DE genes. In the DE group, a notable example of biomarker gene in TNBC, EGFR [56] is present, which suggests the ranking derived by our proposed metric is sensible. The non-DE genes could not have been identified based on the gene expression data alone. However, by integrating the network and gene expression profiles, we could identify these putative biomarker genes that were not differentially expressed.

**Table 2:** The top 20 genes for classifying TNBC patients based on gene ranking metric

**Gene name**	**M value**	**Differentially expressed**
ABL1	58.5	YES
GRB2	27.7	NO
FYN	26	YES
CSNK2B	24.3	YES
NCK1	17.6	YES
TRAF2	15.1	YES
TGFBR1	12.3	NO
MDFI	12.2	NO
EGFR	11.9	YES
ATXN1	11.5	NO
SMAD1	11.3	NO
CCDC85B	11.2	NO
UBQLN4	10.9	NO
PRKCA	10.6	YES
CHD3	10	YES
CRK	9.8	NO
FXR2	9.7	YES
PIK3R1	9.7	YES
EP300	9.5	YES
MAPK6	9.5	NO

For the non-DE genes in Table 2, there have been literatures reporting TGFBR1 and SMAD1 signaling pathways to be related to breast cancer [57,58]. Previous studies also showed MAPK signaling pathway to be activated in triple-negative breast cancer [59]. Gene Ontology (GO) and KEGG pathway enrichment analysis for the top 100 genes by their *M* values was carried out using the DAVID tool [60]. The identified genes were highly enriched in cancer processes or pathways (Additional file 4: Table S1). We further investigated the network degree distribution for the 100 genes. The results showed that these genes tend to be higher degree nodes in the large PPI network (Additional file 5: Figure S4). Our results demonstrated that disease-associated genes have significantly higher connectivity in the PPI network. Similar conclusions have also been reported in the literature [61,62].

EgoNet can be viewed as a feature selection technique that identifies sets of genes to build a predictive model. Specifically, the gene sets considered are an ‘ego’ and its neighboring genes that can be reached from the ego at a certain path length. We leveraged the EgoNet method to search for subnetworks that can distinguish triple negative breast cancer tumors from other breast cancer subtypes, recovering several known breast cancer-related genes. Importantly, our results revealed a list of novel candidate genes that may provide a deeper understanding in breast cancer studies.

## Conclusions

In this study, we proposed EgoNet, an algorithm for selecting subnetworks whose gene expression is predictive of a disease phenotype. The key advantage of EgoNet is its capability to discover potential markers that are not differentially expressed, but are functionally associated with many differentially expressed genes. EgoNet is a general framework for ego-network selection. In this study, we paired EgoNet with SVM to solve a two-class (case/control) decision problem. However, when paired with an appropriate machine learning approach, EgoNet can be readily applied to datasets with continuous, multi-class, and survival outcome variables.

## Methods

### EgoNet algorithm

The EgoNet algorithm is described in the following quasi-code.

### Accessing the significance of the identified ego-network

When an ego-network is identified, a test of significance is performed to obtain the statistical significance. The null distribution of classification accuracy is derived by randomly permuting the phenotypic labels *B* times and calculating the score from the same ego-network each time. The actual score of this ego network is then indexed on the null distribution to obtain a p-value (Figure 2).

### Computation of ego-network node importance

We employ Random Forest to rank the importance of variables, in this case, the importance of nodes of an ego-network for making disease outcome predictions. The relative importance (RI) of a predictor in a Random Forest model is obtained by the out-of-bag (OOB) error estimation, which is the increase of mean squared error (MSE) when the predictor values are permuted.

For each tree *t*, let *OOB*_*t*_ be the associated sample and *errOOB*_*t*_ be the error of *t* on this *OOB*_*t*_ sample. Randomly permute the value of predictor *X*^*j*^ in *OOB*_*t*_ to get a perturbed sample denoted by $\mathrm{OO}B_{t}^{j}$ and compute $\mathrm{err}{\tilde{\mathrm{OOB}}}_{t}^{j}$. The variable importance score of predictor *X*^*j*^ is derived by

$$\mathrm{VI}\left(X^{j}\right)=\frac{1}{T}\sum_{t}\left(\mathrm{err}\tilde{\mathrm{OO}B_{t}^{j}}‒\mathrm{errOO}B_{t}\right)$$

Where T is the number of trees. We used the Python package “sklearn” to implement this procedure.

### The design of simulation study

We simulated each scenario 100 times. In each simulation, we generated a scale-free undirected and no-self-loop network with 500 nodes. Together with the network data, a gene expression dataset with 500 genes and 100 samples was generated by random sampling the expression values from the standard normal distribution. An ego-network is selected by first randomly selecting a node as ego with its network degree between 5 and 20, and then taking the level 1 ego-network from the selected ego node. Eighty percent of the nodes in the ego-network were marked as disease genes, and the phenotypic outcomes were generated based on the expression values of those disease genes using linear and nonlinear models. The linear relationship was formulated as *Y* = ∑ *X*_*i*_, while the nonlinear relationship was formulated as *Y* = ∑ *X*_*i*_^3^. Finally, *Y* was dichotomized to 0 if Y < 0 or 1 if Y ≥ 0.

### Availability

The EgoNet algorithm is implemented by Python scripts and available at https://github.com/cauyrd/EgoNet.

## Abbreviations

TNBC: Triple negative breast cancer; PPI: Protein-protein interaction; RF: Random forest; SVM: Support vector machine; KNN: K-nearest neighbor; FDR: False discovery rate; DE: Differentially expressed; MSE: Mean square error; RI: Relative importance; OOB: Out of bag.

## Competing interests

The authors declare that they have no competing interests.

## Authors’ contributions

RY, ZQ and TY conceived and designed the study. RY implemented the method and conducted the simulation study. RY and TY conducted the data analysis. RY and YB interpreted the biological results. RY, YB and TY wrote the manuscript. All authors read and approved the final manuscript.

## Supplementary Material

Additional file 1: Figure S1 Classification performance (A) and proportion of ego node coverage (B) for the proposed EgoNet method and Chuang *et al.*’s method in different simulation settings. Click here for file

Additional file 2: Figure S2 The distribution of ego-network levels of the identified subnetworks. Click here for file

Additional file 3: Figure S3 Boxplots of the expression levels of some important genes. Click here for file

Additional file 4: Table S1 Enriched GO and KEGG categories for the top 100 disease-associated genes ranked by *M* value. Click here for file

Additional file 5: Figure S4 Network degree distribution of the top 100 identified disease-associated genes ranked by *M* value (red curve) and all genes from the human PPI network (blue curve). Click here for file
