# Supplementary figures and images for: EgoNet: identification of human disease ego-network modules
Source: BMC Genomics. 2014 Apr 28;15:314. doi: 10.1186/1471-2164-15-314 (PMC4234496; doi:10.1186/1471-2164-15-314)

A

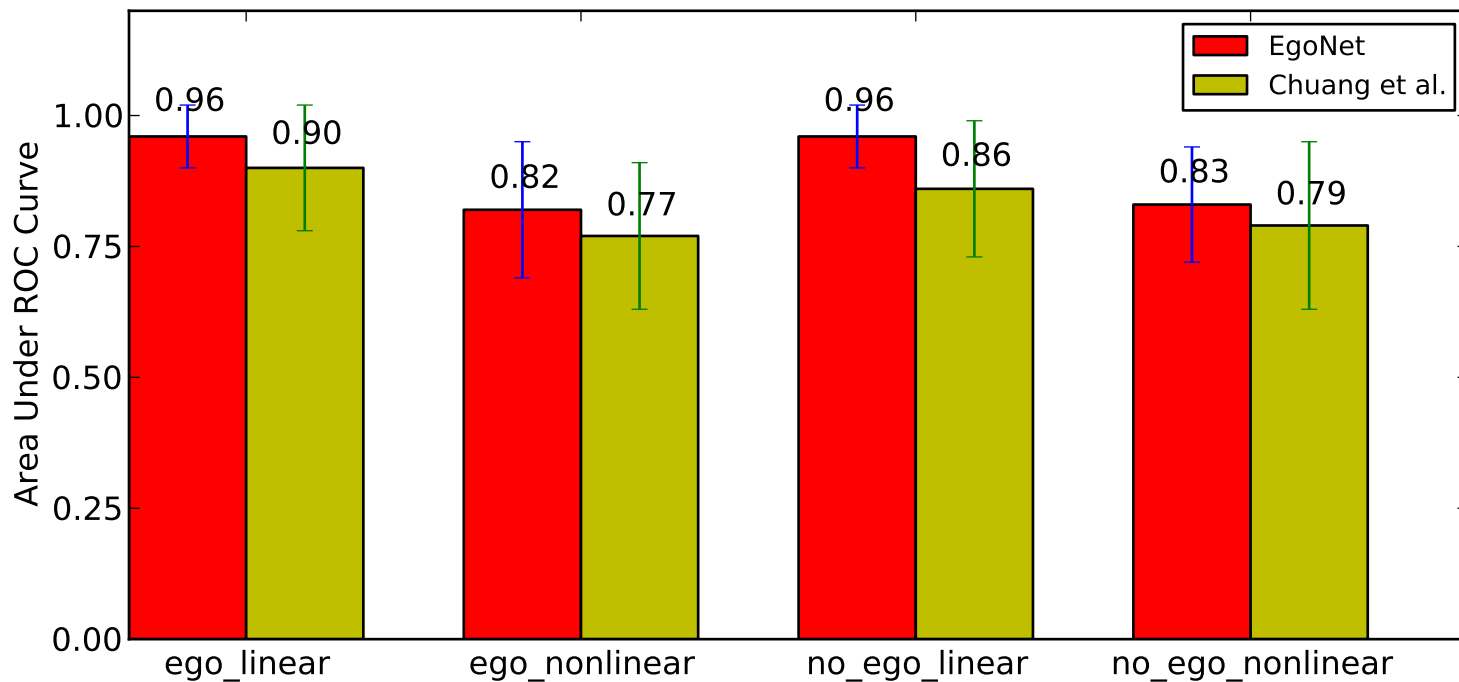

B

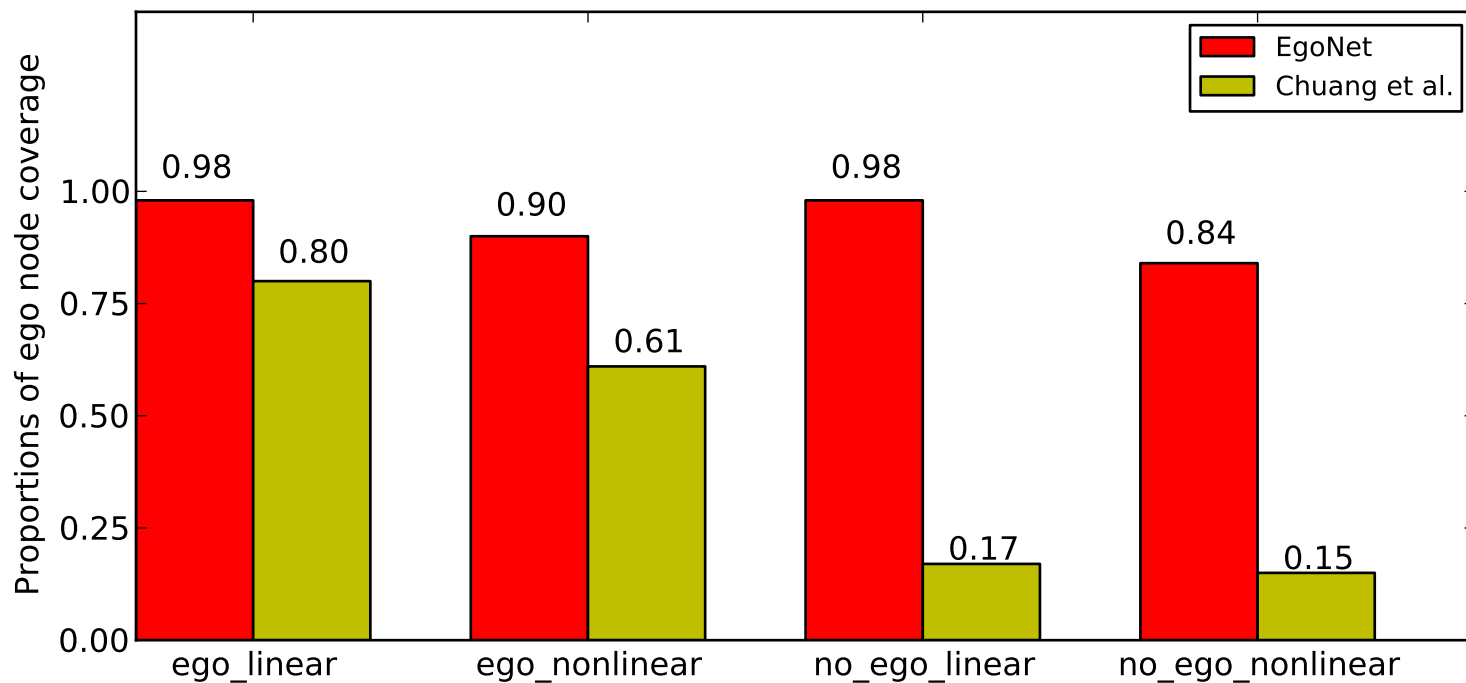

Supplement: Additional file 1: Figure S1 — Classification performance (A) and proportion of ego node coverage (B) for the proposed EgoNet method and Chuang et al.’s method in different simulation settings. [file 1471-2164-15-314-S1.pdf]

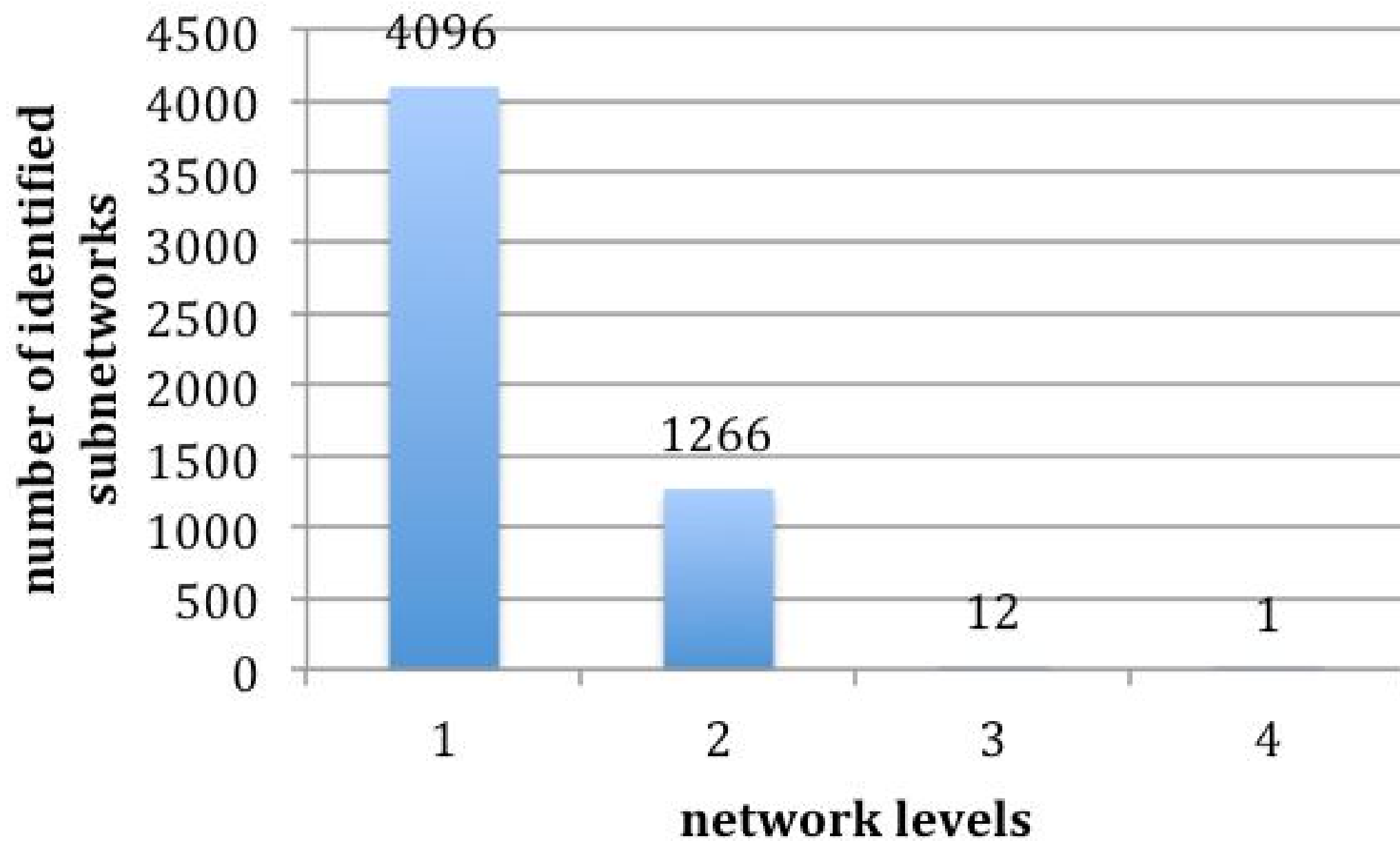

Supplement: Additional file 2: Figure S2 — The distribution of ego-network levels of the identified subnetworks. [file 1471-2164-15-314-S2.pdf]

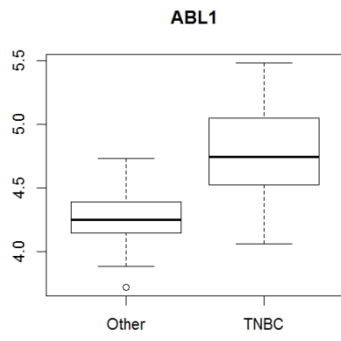

(a)

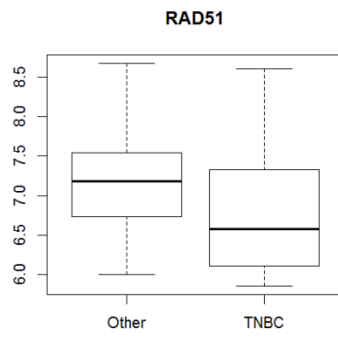

(b)

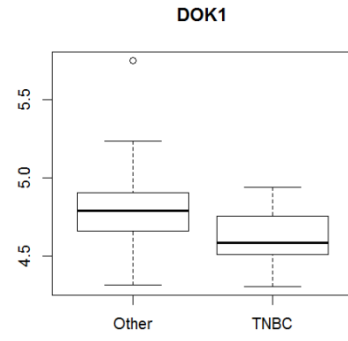

(c)

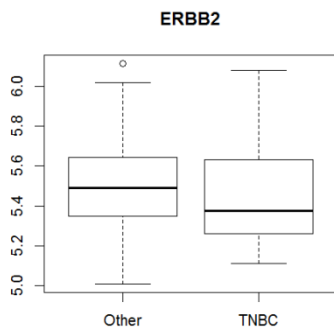

(d)

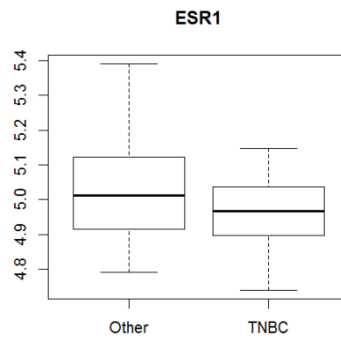

(e)

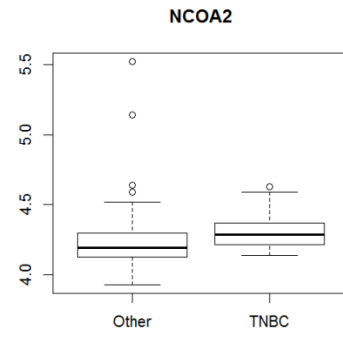

(f)

Figure S3. The expression levels of some important genes.

Supplement: Additional file 3: Figure S3 — Boxplots of the expression levels of some important genes. [file 1471-2164-15-314-S3.pdf]

Degree rank plot

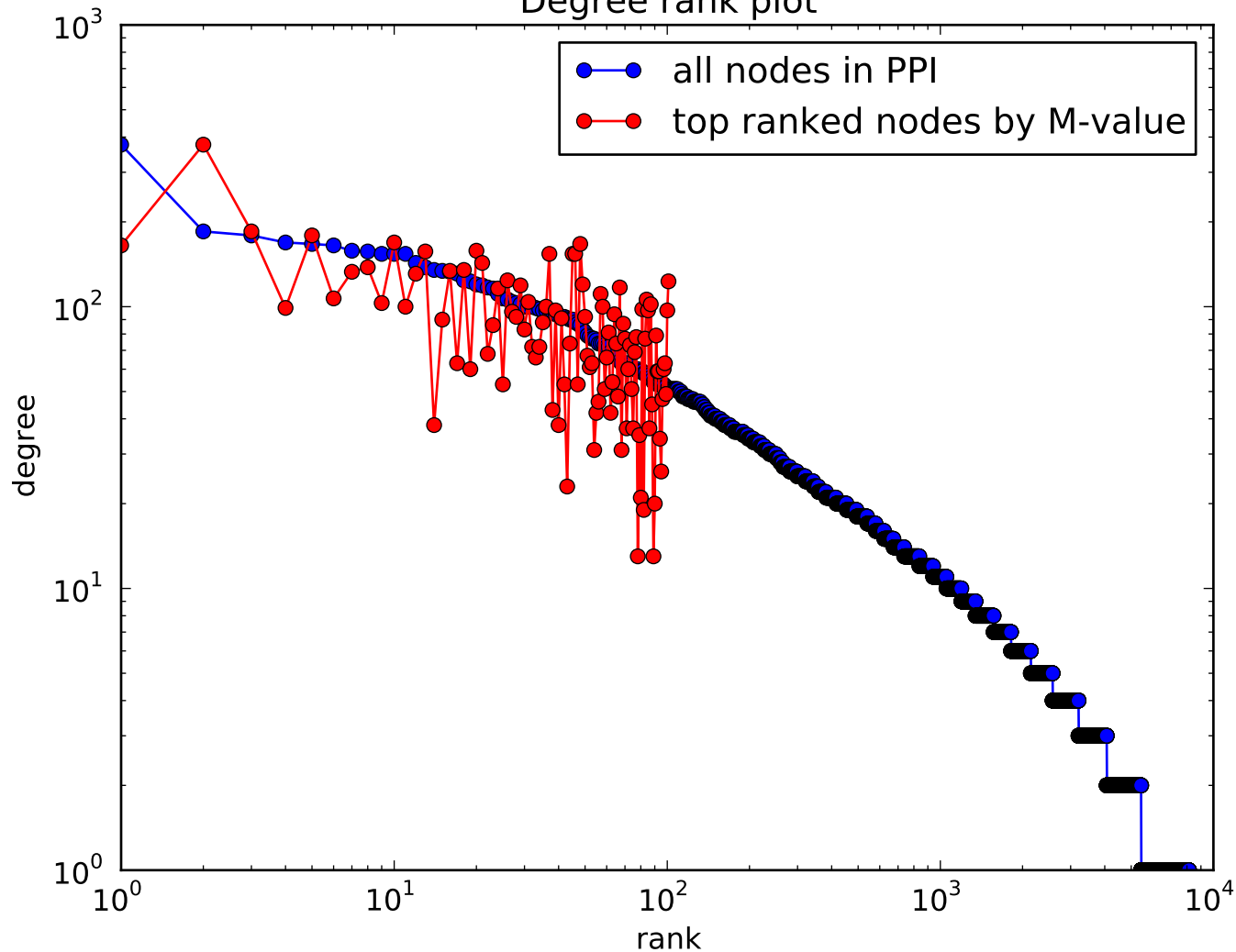

Supplement: Additional file 5: Figure S4 — Network degree distribution of the top 100 identified disease-associated genes ranked by M value (red curve) and all genes from the human PPI network (blue curve). [file 1471-2164-15-314-S5.pdf]
